# Magnetic resonance enterography in pregnant women with Crohn’s disease: case series and literature review

**DOI:** 10.1186/1471-230X-14-146

**Published:** 2014-08-16

**Authors:** Myriam D Stern, Uri Kopylov, Shomron Ben-Horin, Sarah Apter, Marianne Michal Amitai

**Affiliations:** 1 Department of Diagnostic Imaging, Sheba Medical Center, Ramat Gan, Israel; 2 Department of Gastroenterology, Sheba Medical Center, Ramat Gan, Israel

**Keywords:** Crohn’s disease, Pregnancy, Magnetic resonance enterography, MRI

## Abstract

**Background:**

Evaluation of pregnant women with known or suspected Crohn’s disease (CD) remains a challenge. Magnetic Resonance Enterography (MRE) is a promising diagnostic tool in these patients; however, the clinical data on MRE utilization in pregnancy is scarce. The aim of the study was to describe the experience with MRE in pregnant CD patients in a tertiary referral center.

**Methods:**

We retrospectively reviewed MRE studies performed in pregnant women with known or suspected CD that were performed between January 2007 and November 2012. Imaging findings, clinical management and outcome were extracted from patient’s file and electronic records. Image quality was evaluated.

**Results:**

Ten studies of 9 patients were included. MRE protocol was modified to maximize maternal and fetal safety, and intravenous gadolinium was not used. In 7 patients, CD diagnosis was previously established; six were admitted with clinical symptoms consistent with CD exacerbation, and an additional patient with a recurrent groin abscess without apparent luminal symptoms. In all seven patients, imaging features consistent with active CD were detected; new penetrating complications were detected in 4 patients. Two patients underwent MRE for suspected CD which was not comforted by study results. The clinical management was significantly impacted by MRE results in all positive cases. The image quality of the fast MRE sequences obtained without gadolinium was satisfactory and allowed meaningful interpretation.

**Conclusion:**

MRE with an adapted protocol for pregnancy is a reliable imaging modality to manage in pregnant women with known or suspected CD.

## Background

Pregnant women with Crohn’s disease (CD) pose a range of diagnostic and clinical challenges [1]. When a pregnant woman presents with a clinical picture consistent with suspected new-onset CD or exacerbation of previously established CD, diagnostic options are somewhat limited due to maternal and fetal safety concerns. Endoscopy can be performed if needed for evaluation of colonic or distal ileal inflammation with necessary procedural precautions and careful selection of a sedation regimen, however, evaluation of small-bowel disease and penetrating complications usually requires cross-sectional imaging [2]. Prompt evaluation and management are required, as active CD or penetrating complications carry a significant risk of morbidity such as preterm delivery and low birth weight [3-6]. Accurate anatomical information and evaluation of CD complications is mandatory in considering and planning surgical intervention, when necessary, in severely ill patients. Moreover, imaging may be valuable in planning the mode of delivery, as cesarean section is usually recommended in patients with active perianal disease [5,7].

Both computer tomography enterography (CTE) and magnetic resonance enterography (MRE) are commonly used to evaluate disease activity and complications in CD patients. However, CTE is associated with a significant exposure to ionizing radiation potentially harmful for the fetus [8,9].

MRE is not associated with radiation exposure and therefore is the preferred cross-sectional diagnostic modality in pregnancy. However there is a paucity of data outlining the utilization of MRE in pregnant CD patients. In particular, safety data pertaining to the intravenous contrast material (gadolinium) routinely utilized in MRE is very limited, and it’s use in pregnancy recommended only when absolutely necessary [10].

Several typical MRE findings reflect disease activity in CD [11]. Bowel wall thickness, relative contrast enhancement, presence of edema, and mucosal ulcerations have been shown to be independently correlated with endoscopic disease activity. An index incorporating these parameters (Magnetic resonance index of activity, MaRIA) was significantly correlated with the Crohn’s Disease Endoscopic Index of Severity (CDEIS) [12].

An additional MRE scoring system based on mural thickness and T2 signal intensity was demonstrated to be significantly correlated with a histological score of the terminal ileum inflammation [13].

The purpose of the present study is to report our experience in with MRE utilizing a modified protocol adapted to maximize patient and fetus safety in the context of suspected CD or CD exacerbation in a tertiary referral center setting.

## Methods

### Study population

The study cohort included all pregnant patients who underwent MRE studies for suspected or established CD between 01/2007-12/2012. Relevant demographic and clinical data was retrieved from the patient’s files and electronic records. This retrospective study was approved by ethics review board of Chaim Sheba medical center.

### MRE protocol

All MRE studies were performed on a 1.5 T MR System (General Electric Healthcare, Little Chalfont, Buckinghamshire, United Kingdom) with an 8 channels cardiac coil. Our standard MRE protocol was adapted for pregnant patients. Oral contrast with Manitol 5% (1000 ml) was administered 60 minutes prior to the examination. However, neither Gadolinium chelate nor glucagon (used to minimize bowel motility) were administered.

The obtained MRE sequences included:

FIESTA (Fast Imaging Employing Steady State Acquisition) in axial, coronal and sagittal planes (repetition time (TR)/echo time (TE) = 4.2-5/1.2-2.3 milliseconds (ms), slice thickness 6 mm, without interslice gap, field of view (FOV) of 36–44 cm^2^, acquisition matrix of 384×384, number of excitations (NEX) o = 1; flip angle 60°):

FSE (Fast spin Echo) T2 with fat suppression, in axial and coronal planes (TR/TE =1555-3500/88-93 ms, slice thickness 6 mm without interslice gap, FOV of 44 cm^2^, acquisition matrix of 384×224, NEX =1):

T1 weighted 2D FSPGR (Fast spoiled Gradient Recalled Acquisition in the Steady State) with fat suppression and breath hold in axial and coronal planes (TR/TE = 75-155/1.3-4.2 ms, slice thickness 6 mm, without interslice gap, FOV of 36 cm^2^, acquisition matrix of 512×256, NEX =1, flip angle 75°).

T1 weighted 3D LAVA (Liver Acquisition with Volume Acceleration) (without contrast) with fat suppression and breath hold in coronal and axial planes (TR/TE = 3.6-4.3/1.7-2.1 ms, coronal: slice thickness 4 mm, no interslice, axial: slice thickness 6 mm and overlap of 3 mm, FOV of 44 cm^2^, acquisition matrix of 416-320*192, NEX =0.75, flip angle 15°).

### Analysis of MRE findings

MRE studies were reviewed by two experienced abdominal radiologists (MMA, SA) for signs of active and chronic CD and extraluminal complications. The main recorded findings included:

*Mural findings:* mural thickening of 3 mm or more, ulcerations, wall edema (high T2 signal on FSE sequence), luminal stenosis, prestenotic dilatation.

*Mesenteric findings*: congestion (comb sign), hypertrophy (creeping fat), and lymphadenopathy. (lymph node shortest diameter > 10 mm).

*Extra luminal findings*: phlegmon, abscess, fistula, free fluid.

Bowel obstruction, bowel perforation or acute hemorrhage were considered as findings requiring urgent surgical attention.

### Image quality assessment

The image quality of each sequence for every MRE study was evaluated using a score varying from poor to good (1–3): 1 = blurred image, 2 = fairly satisfactory image, 3 = clear image. A mean score was obtained for each sequence by both reviewers.

The clinical course and outcomes of the included patients was described.

## Results

The study population included nine pregnant CD patients who underwent a total of ten MRE studies. The indications for MRE studies were as follows:

Clinical exacerbation of known CD (n = 7), including exacerbation of luminal disease in 6 patients (in one accompanied by a new-onset cholestasis and in another one- by preeclampsia) and a recurrent groin abscess in one patient; suspected CD (n = 2).

The demonstrated MRE findings for each patient are described in detail in Table 1. In all patients with known CD, pathognomonic signs of active disease were demonstrated on MRE. Wall thickening and ulcers were demonstrated in 6 patients (86%), high T2 signal in bowel wall in 4/4 patients who had T2 sequences performed, phlegmon in 4/7 (57%) and abscess in 1/7 (14%). Positive comb sign (a hallmark of mesenteric inflammation) was demonstrated in all patients. Fistulae were seen in 3 (43%), while stenosis and prestenotic dilatation were seen in 5 (71%).

**Table 1:** Summary of MRE findings in pregnant patients with known (1–7) or suspected CD (8, 9), NA: not available

**Patient number**	**1a**	**1b**	**2**	**3**	**4**	**5**	**6**	**7**	**8**	**9**
**Mural signs**										
Small bowel mural thickening	+	+	-	-	+	-	+	+	-	-
Large bowel mural thickening	+	+	+	-	-	+	+	+	-	+
Mural high T2 signal	+	+	NA	NA	+	NA	+	+	-	NA
Stenosis & prestenotic dilatation	+	+	-	-	+	+	+	+	-	+
Ulcers	+	+	+	-	+	+	+	+	-	+
**Mesenteric signs**										
Comb sign	+	+	+	+	+	+	+	+	-	-
Creeping fat	+	+	+	+	+	+	+	-	-	-
Lymphadenopathy	-	-	+	-	+	-	+	+	+	-
**Complications**										
Phlegmon	+	+	-	-	+	-	+	+	-	-
Abscess	-	+	-	-	-	-	-	-	-	-
Fistula	++	++	-	-	+	-	+	-	-	-
Free fluid	-	-	-	-	-	-	+	-	-	-

**Figure 1:**
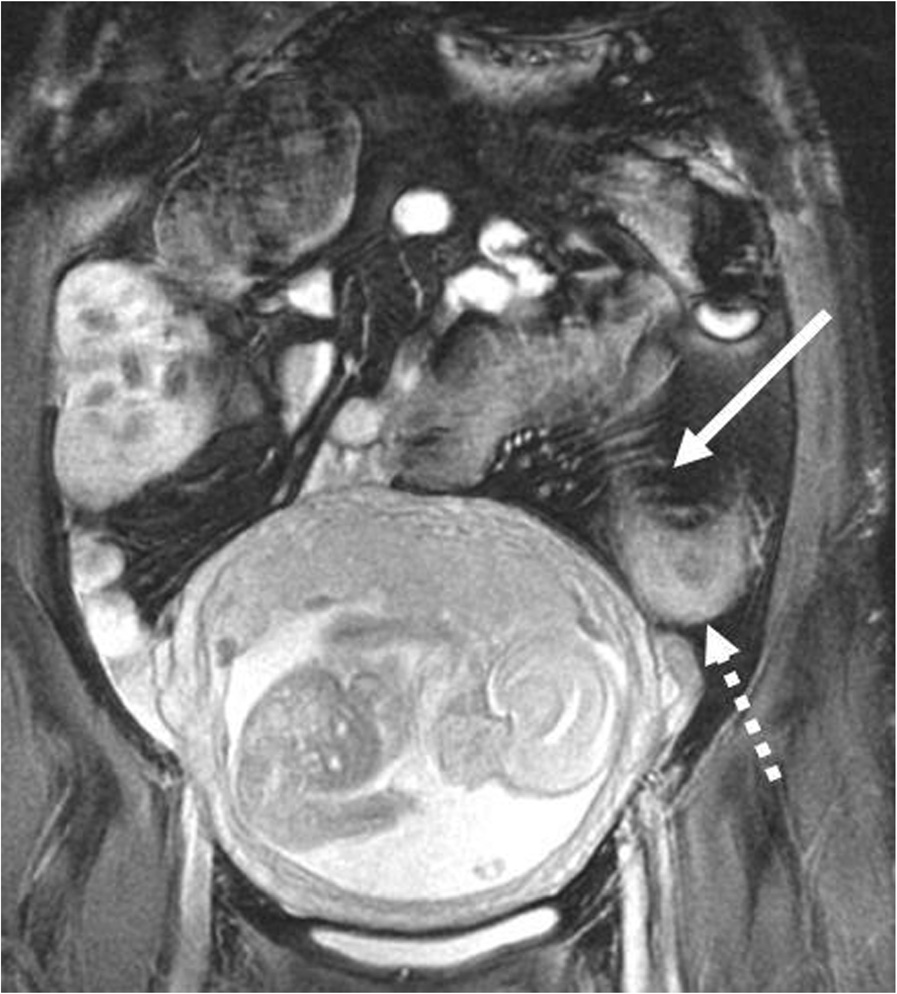
A 19 weeks pregnant patient with CD, Coronal FIESTA: signs of active disease: mesenteric congestion (arrow) and large bowel mural thickening and edema (dotted arrow).

**Figure 2:**
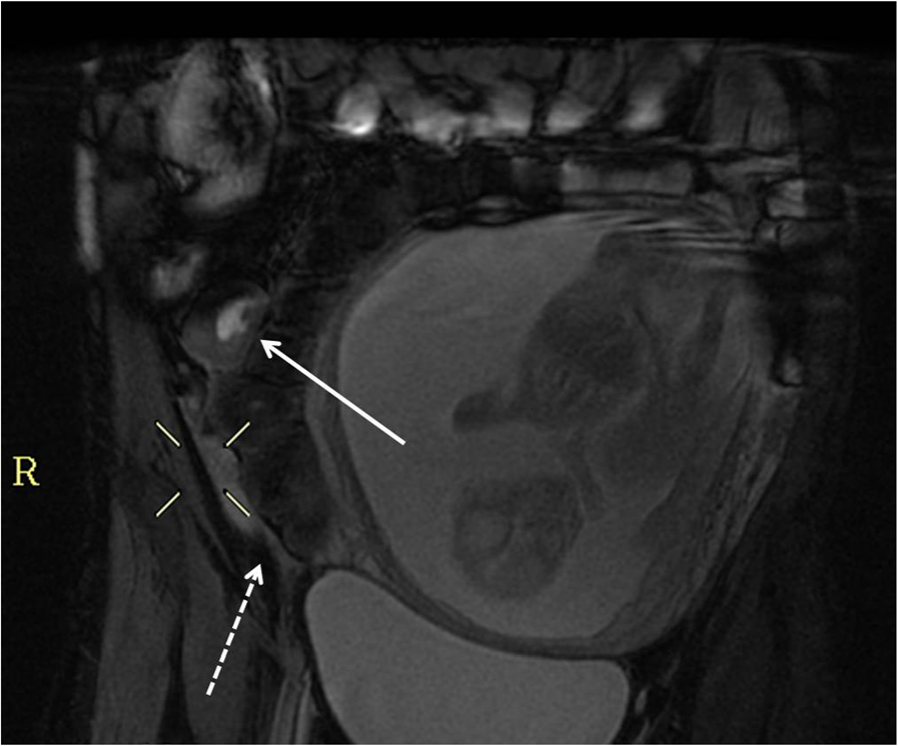
A 20 weeks pregnant patient with CD, Coronal FIESTA: signs of active disease: small bowel mural thickening and ulcer (arrow), note free fluid (dashed arrow).

In two patients, MRE was performed for clinical suspicion of CD. In one patient, no radiological findings compatible with this diagnosis were demonstrated. The second patient was diagnosed with ulcerative colitis in the past. Her study demonstrated colonic findings compatible with UC and not CD (large bowel mural thickening, submucosal edema).The typical radiographic signs of active CD identified on our modified protocol for pregnancy included mural thickening of 3 mm or more, ulcers, wall edema, comb sign, phlegmon, abscess and fistula are presented in Figures 1 and 2.

### Image quality assessment

FIESTA sequences were of very good quality and enabled accurate assessment of the bowel anatomy, as well as the presence of a phlegmon, an abscess or free fluid. The images did not have significant motion artifacts.

Fast spin echo T2 sequences, although not available for all patients, were useful for evaluation of edematous bowel loops and in ruling out the presence of free fluid. Gradient echo (GRE) T1 weighted 3D LAVA sequences were blurred by motion artifacts. GRE T1 weighted 2D FSPGR had motion artifacts and their added diagnostic value was relatively low. Coronal planes were easier to interpret than axial planes (Table 2).

**Table 2:** Sequences quality score of MRE protocol adapted to pregnancy

**MRE sequence**	**Quality score**
FIESTA coronal	2.8
FIESTA sagittal	2.8
FIESTA axial	3
Fast SE T2 coronal	2.8
Fast SE T2 axial	2.2
FSPGR 2D T1 coronal + fat sat	1.6
FSPGR 2D T1axial + fat sat	1.5
LAVA 3D T1 coronal + fat sat	1.6
LAVA 3D T1 axial + fat sat	2

FIESTA- Fast Imaging Employing Steady State Acquisition.

SE-spin echo.

FSPGR- Fast spoiled Gradient Recalled Acquisition in the Steady State.

LAVA- Liver Acquisition with Volume Acceleration.

### Clinical impact of the MRE findings

**Figure 3:**
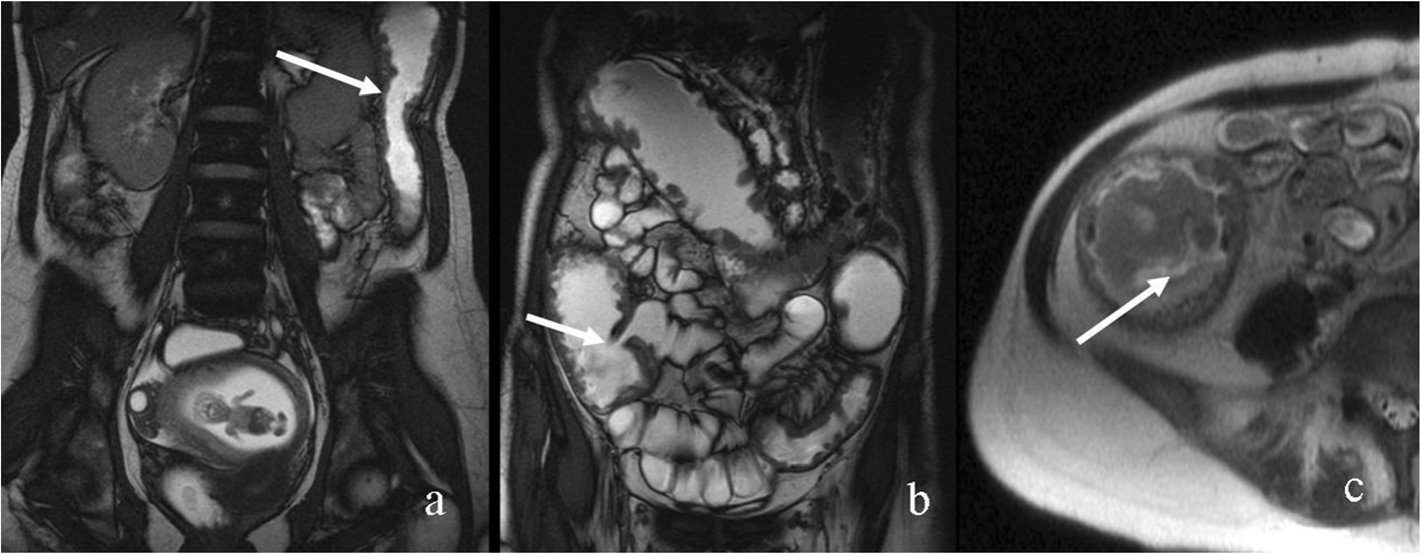
A 11 weeks pregnant patient diagnosed with UC, a: Coronal FIESTA, large bowel mural thickening with a thumb printing pattern (arrow), b: Coronal FIESTA, ileo-cecal stenosis (arrow), c: Axial SSFSE T2 submucosal edema (arrow).

In all the 9 patients, MRE findings have contributed useful clinical information and impacted the clinical management (Table 3). The findings of the MRE were valuable in ruling out the diagnosis in the two patients suspected of CD: one patient (n°8) had no radiological findings suggesting CD and did not require anti-inflammatory treatment. In the other patient, the diagnosis of UC was confirmed and CD was ruled out, as no signs of small bowel or mesenteric disease and no penetrating complications were demonstrated (Figure 3).

**Table 3:** Indications, MRE findings and clinical outcome of pregnant CD patients

**Patient number**	**CD status prior to pregnancy**	**Pregnancy number/week**	**Indication for MR**	**Principal MRE findings**	**Clinical management**	**Clinical outcome**	**Pregnancy outcome**
1	20 years duration	1/23	Clinical exacerbation of known CD	Phlegmon, sinus tract and fistula	Prednisone therapy and IV antibiotics	No improvement on medical treatment	
	Inflammatory phenotype						
	Ileocolonic distribution						
	No current treatment						
		1/26	Clinical exacerbation	Small abscess 3 weeks later	Abscess not accessible to drainage, conservative treatment with steroids and IV antibiotics	Clinical deterioration, surgical intervention one month post-delivery, including ileostomy and cecectomy.	Spontaneous VD at 34 weeks, healthy newborn
2	9 years duration	2/19	Clinical exacerbation of known CD	Active disease, no complications, no obstruction	Addition of IV steroids	Clinical response and discharge	Spontaneous VD at 38 weeks, healthy newborn
	Inflammatory phenotype						
	Ileocolonic distribution						
	Tx: Azathioprine						
3	16 years duration	1/31	Clinical exacerbation of known CD new onset of cholestasis	Scant signs of active disease, no complications, no obstruction	UDCA and prednisone added to maintenance treatment with 6 MP	Improvement of CD symptoms, persistent cholestasis	Induced preterm vaginal delivery for cholestatsis at 35 weeks healthy newborn
	Fibrostenotic phenotype						
	s/p ileocecectomy						
	Ileocolonic distribution						
	Tx: 6-MP						
4	15 years duration	2/22	Clinical exacerbation of known CD	Active disease phlegmon and fistulae	Enteral nutrition modulation a	Partial response phlegmon and fistulae in CT post- delivery, antibiotics: Adalimumab was after delivery	Spontaneous vaginal delivery at 38 weeks, healthy newborn
	Fibrostenotic and inflammatory phenotype						
	Ileocolonic distribution						
	Tx: Azathioprine						
5	4 years duration	1/37	Clinical exacerbation of known CD preeclampsia	Some signs of active disease, no complications	IV steroids and antibiotics	Preeclampsia Urgent delivery	Spontaneous onset of labor, vaginal delivery converted to C/S, at 37 weeks healthy newborn
	Inflammatory phenotype						
	Inactive perianal disease						
	Crohn’s colitis						
	Tx: infliximab						
6	10 years duration	2/20	Recurrent abscess in right groin, fistula?	Phlegmon in RLQ fistula to right groin	IV and PO antibiotics and abscess drainage prior to MR	Clinical improvement	Spontaneous delivery, healthy newborn at week 38
	Inflammatory phenotype						
	Ileocolonic distribution						
	Tx: Azathioprine						
7	2 years duration	2/25	Clinical exacerbation of known CD	Signs of active disease, new phlegmon in RLQ	Conservative treatment with steroids and IV antibiotics emergency cerclage	Temporary clinical improvement hypoalumiemia & anasarca	Spontaneous vaginal delivery at 28 weeks healthy very low birth weight newborn
	Inflammatory phenotype						
	Ileocolonic distribution						
	Tx: 6-MP and adalimumab						
8	No known disease	1/26	Suspected CD	Bowel normal	No treatment	Abdominal symptoms resolved	Healthy twins newborns C/S at 32 w
9	No known disease	?/11	Uncertain diagnosis of UC, suspected CD	MRE signs of UC	NA	NA	Spontaneous delivery with a healthy newborn at week 41S

UC-ulcerative colitis, VD- vaginal delivery, RQ-right lower quadrant, 6-mp- 6-mercaptopurine, UDCA-ursodeoxycholic acid.

In all patients with known CD, no complications requiring emergent surgical interventions such as obstruction, perforation or hemorrhage were demonstrated. The patients were treated conservatively and did not require surgical intervention prior to the delivery.

**Figure 4:**
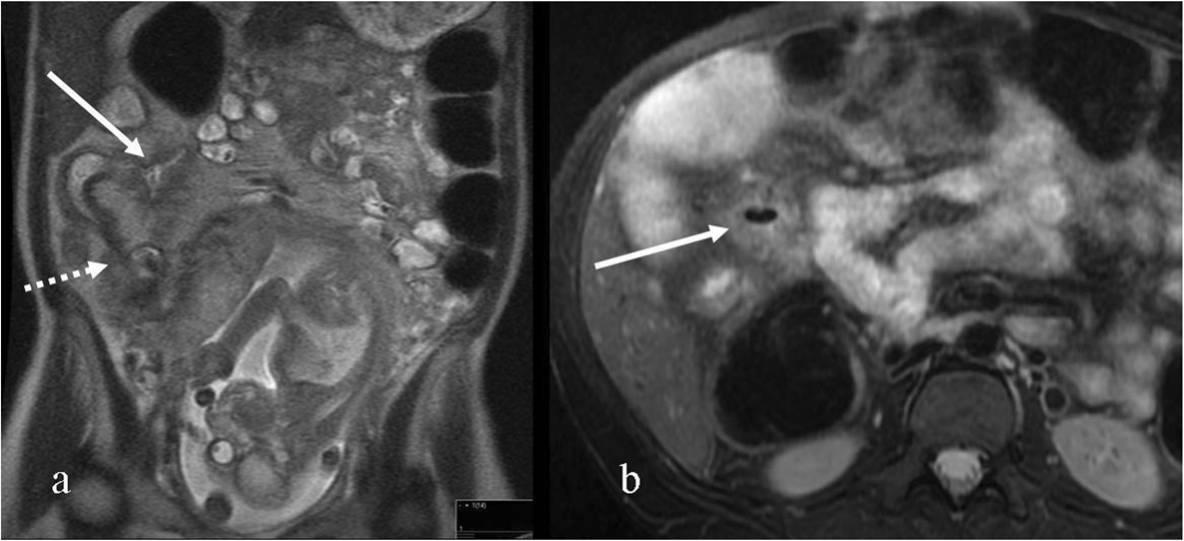
A pregnant patient with CD at 23 weeks (a) and 26 weeks (b) of pregnancy: a: Coronal FIESTA fistula (dotted arrow) and sinus tracts (arrow), b: Axial heavily weighted T2, abscess near confluence of sinus tract in the same patient three weeks later (arrow).

Extraluminal complications such as phlegmon, abscess, fistula, free fluid were ruled out in 3 patients (n°2,3,5). In one patient with preeclampsia (n°5), MRE was performed one day before delivery, and assisted the decision to perform a cesarean section approach since no intra-abdominal complications were detected. In two others (n°2 & 3), medical treatment was adjusted with good response of CD symptoms.In four patients (n°1,4,6,7), extraluminal complications were newly diagnosed by MRE .In one of them (n°1) the visualization of a phlegmon transforming into an abscess, inaccessible for drainage, was detected on a second MRE examination, and impacted a physician decision to undertake a surgical intervention after a spontaneous vaginal delivery at 34 weeks. MRE findings were confirmed during surgery (Figure 4).

In patient n°4, phlegmon and fistula were demonstrated. A partial clinical response was obtained with intravenous antibiotics until spontaneous vaginal delivery at 38 weeks. Due to persistence of the clinical exacerbation after the delivery, an additional post-delivery CT was performed, which confirmed the MR findings.

Patient n°6 presented with a recurrent groin abscess that required multiple drainage procedures, without clinical exacerbation of the luminal disease. An MRE was performed at week 12 of pregnancy. The study demonstrated active disease in terminal ileum and cecum accompanied by a phlegmon and a new inflammatory tract communicating to another phlegmonous subcutaneous zone corresponding to the location of the drained abscess.

In the fourth patient (n°7) who presented with CD exacerbation, cervical widening and early contractions at 17 week of pregnancy, a phlegmon was demonstrated. The patient did not respond to medical treatment, and spontaneus delivery occurred at 28 week. On presentation this patient was treated with a combination of 6-mercaptopurine and adalimumab. There is no clear evidence to suggest that a combination of adalimumab with a thiopurine is superior to adalimumab alone in CD (as opposed to Infliximab where such benefit was clearly demonstrated [14]) and pregnancy-related data are even more limited.

## Discussion

MRE has long been established as an accurate and safe modality for diagnosis of CD, monitoring of disease activity and detection of complications [2]. In our series, it appears that, despite the technical limitations (mainly caused by avoidance of intravenous contrast material and by motion artifacts), MRE was able to demonstrate significant and clinically relevant findings impacting the clinical management in pregnant patients. Important classical stigmata of CD were demonstrated and confirmed on subsequent studies in some of the patients; non-emergent surgical procedures were deferred until after delivery and additional clinical decisions such as mode and timing of planned delivery were impacted by MRE. Thus, the information provided by MRE plays a key role in the clinical and surgical management of the patients.

**Figure 5:**
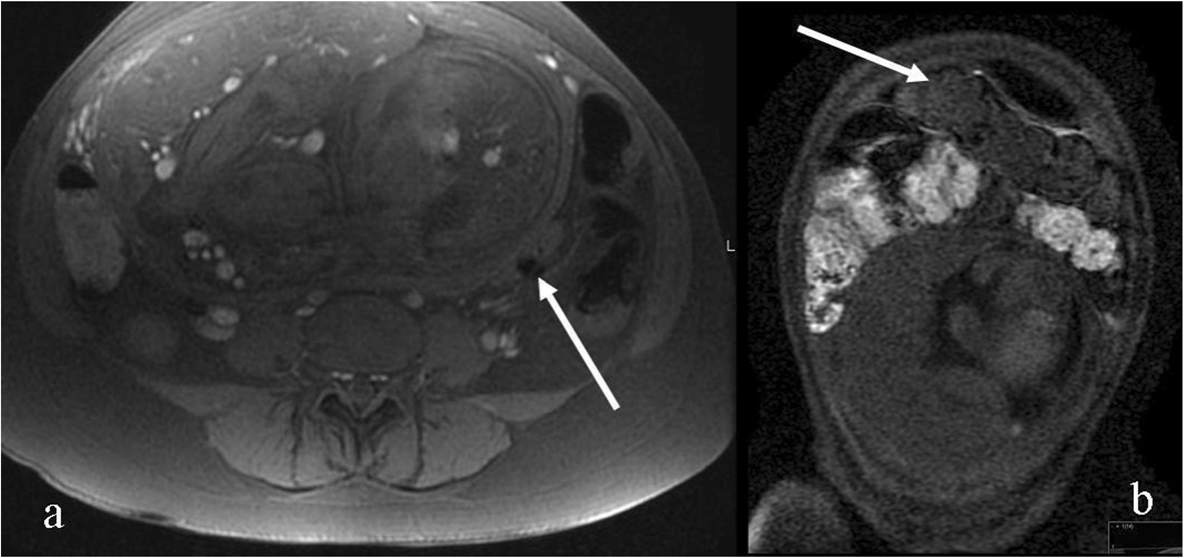
The impact of an enlarged uterus; a: a 37 weeks pregnant CD patient: Axial FSPGR 2D, pseudo stenosis caused by compressing uterus, b: a 26 weeks pregnant patient: Coronal LAVA, sigma displaced cranially (not to be misinterpreted as transverse colon above) (arrow).

To the best of our knowledge, our study is the largest case series describing the utility of MRE in pregnant CD patients published to date. In addition, in our series we have utilized oral contrast material, which has not been reported in pregnant CD patients in current literature. In an early article, Shoenut et al. [15] reported two cases of new onset of CD during pregnancy diagnosed by MR without oral contrast material but with Gadolinium injection. Mural thickening and enhancement of the terminal ileum were consistent with CD and subsequently histologically confirmed [15]. Other studies have been reported where MR imaging was used for the triage of pregnant women presenting with acute abdominal pain. Oto et al. described their experience with MR imaging of 4 pregnant patients with known CD and exacerbation [16]. The advantage of gadolinium-enhanced MR imaging in pregnant patients with acute abdominal pain was also reported by Bichard and colleagues in one case of CD in a series of 29 pregnant patients with acute abdominal pain showed free fluid in the abdomen, thickening of bowel walls, and diffuse enhancement of the peritoneum [17]. In contrast to our studies, these reports have focused on patients with emergent clinical presentations, and employed different protocols aimed at detection of urgent surgical complications , as opposed to a dedicated MRE protocol that is optimized for evaluation of signs of luminal and extraluminal disease activity in CD. Moreover, in the described studies CD patients were a minority within a patient cohort that included patients evaluated for multiple urgent indications.Interpretation of MRE in pregnancy presents particular challenges. The first difficulty is related to anatomical changes caused by the enlarged uterus, such as pseudo-stenosis and bowel loop displacement. The sigmoid colon may appear stenosed due to pressure of the uterus (see Figure 5a). Another possible anatomical change is the upward move of the sigmoid colon cranial to the uterus as presented in Figure 5, where the sigma was initially misinterpreted as the transverse colon.

**Figure 6:**
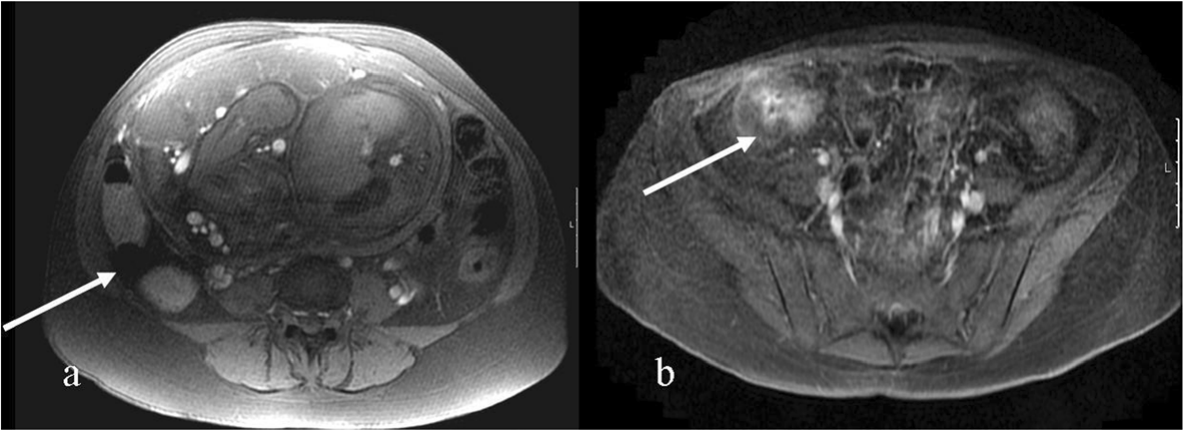
A 37 weeks pregnant patient a: Axial FSPGR, wall thickening of terminal ileum overlooked (arrow) but retrospectively seen by comparing with previous MRE a year earlier, b: Axial LAVA post Gadolinium injection (arrow) same patient a year earlier.

An additional challenge stems from a significant decrease in image quality due to motion artifacts caused by fetal movements, difficulty in breath holding and bowel motion resulting from the omission of glucagon injection in the modified protocol. Motion artifacts may be partially overcome with fast acquired sequences such as FIESTA which produced the highest quality images in our study. An additional complexity results from non-utilization of gadolinium, preventing the clear demonstration of the wall enhancement. For instance, in Figure 6a the wall thickening, initially overlooked, was seen only after comparison with a previous MRE study (Figure 6b) with gadolinium injection performed prior to pregnancy. The use of gadolinium in pregnancy is debatable. Although no adverse effects to the fetus have been documented with gadolinium infusion, the American College of Radiology recommends its use only when there is an outstanding benefit [10].

Nevertheless, we administer an oral contrast agent (mannitol 5%) as normally used in enterography and enteroclysis, which we believe to be essential for evaluating bowel loop anatomy. Mannitol is a pregnancy class C agent, with no evidence of a detrimental effect on fetal health and development.

The limitation of this study is the small patient group and the retrospective nature of the study. Nevertheless, despite the fact that MRE examinations were performed in sub-optimal conditions with an adapted MRE protocol maximizing maternal and fetal safety, the results were clinically useful and accurate .Our results can also be compared to recent studies using diffusion weighted sequences that provide adequate visualisation of the affected bowel loops in pediatric patients activity without the need for intravenous administration of contrast medium [18]. Diffusion weighted sequences are currently performed in pregnant women for fetal or abdominal evaluation, although they are prone to movement artifacts and their safety has not been evaluated in pregnancy; these sequences were not performed in our standard protocol but could be added in future evaluation.

Radiological limitations did not hamper the clinical utility of our MRE studies, which proved to be very useful in managing the patients and also concurred with the clinical outcome. Nonetheless, further larger studies are pertinent in order to establish the exact role of MRE in the management of Crohn’s patients during pregnancy.

## Conclusion

In conclusion, modified MRE sequencing provided accurate and clinically meaningful results in our series. MRE studies with an adapted protocol for pregnancy are useful in establishing disease activity and diagnosis of complications in pregnant patients with Crohn’s disease and helped to rule out CD in pregnant patients.

## Abbreviations

MRE: Magnetic resonance enterography; CD: Crohn’s disease; UC: Ulcerative colitis.

## Competing interests

The authors declare that they have no competing interests.

## Authors’ contributions

MDS conceived of the study, and participated in its design, carried out the studies, data analyses and drafted the manuscript. SB provided and drafted medical data. UK drafted and reviewed the manuscript. SA participated in the design of the study and helped to draft the manuscript. MMA conceived of the study, coordinated it, participated in its design, provided imaging data and analysis and helped to draft the manuscript. All authors read and approved the final manuscript.

## Pre-publication history

The pre-publication history for this paper can be accessed here:

http://www.biomedcentral.com/1471-230X/14/146/prepub
